# The Homogeneous Azorean Machado-Joseph Disease Cohort: Characterization and Contributions to Advances in Research

**DOI:** 10.3390/biomedicines11020247

**Published:** 2023-01-18

**Authors:** Manuela Lima, Mafalda Raposo, Ana Ferreira, Ana Rosa Vieira Melo, Sara Pavão, Filipa Medeiros, Luís Teves, Carlos Gonzalez, João Lemos, Paula Pires, Pedro Lopes, David Valverde, José Gonzalez, Teresa Kay, João Vasconcelos

**Affiliations:** 1 Faculdade de Ciências e Tecnologia, Universidade dos Açores, 9500-321 Ponta Delgada, Portugal; 2 Instituto de Biologia Molecular e Celular (IBMC), Universidade do Porto, 4200-135 Porto, Portugal; 3 Instituto de Investigação e Inovação em Saúde (i3S), Universidade do Porto, 4200-135 Porto, Portugal; 4 Serviço de Psicologia Clínica, Hospital do Divino Espírito Santo, 9500-370 Ponta Delgada, Portugal; 5 Unidade de Psicologia Clínica, Hospital do Santo Espírito da Ilha Terceira, 9700-049 Angra do Heroísmo, Portugal; 6 Serviço de Neurologia, Hospital do Santo Espírito da Ilha Terceira, 9700-049 Angra do Heroísmo, Portugal; 7 Serviço de Neurologia, Hospital do Divino Espírito Santo, 9500-370 Ponta Delgada, Portugal; 8 Serviço de Patologia Clínica, Unidade de Saúde da Ilha das Flores, 9500-370 Santa Cruz das Flores, Portugal; 9 Augenarztpraxis Petrescu Wuppertal, Department of Ophthalmology, 42389 Wuppertal, Germany; 10 Serviço de Genética Médica, Hospital D. Estefânia, 1169-045 Lisboa, Portugal; 11 Hospital Internacional dos Açores (HIA), 9560-421 Ponta Delgada, Portugal

**Keywords:** MJD, SCA3, spinocerebellar ataxia type 3, homogeneous cohorts, European Spinocerebellar Ataxia type 3/Machado-Joseph disease Initiative (ESMI), polyglutamine disorders, clinical trials

## Abstract

Machado-Joseph disease (MJD)/spinocerebellar ataxia type 3 (SCA3) is the most common autosomal dominant ataxia worldwide. MJD is characterized by late-onset progressive cerebellar ataxia associated with variable clinical findings, including pyramidal signs and a dystonic-rigid extrapyramidal syndrome. In the Portuguese archipelago of the Azores, the worldwide population cluster for this disorder (prevalence of 39 in 100,000 inhabitants), a cohort of MJD mutation carriers belonging to extensively studied pedigrees has been followed since the late 1990s. Studies of the homogeneous Azorean MJD cohort have been contributing crucial information to the natural history of this disease as well as allowing the identification of novel molecular biomarkers. Moreover, as interventional studies for this globally rare and yet untreatable disease are emerging, this cohort should be even more important for the recruitment of trial participants. In this paper, we profile the Azorean cohort of MJD carriers, constituted at baseline by 20 pre-ataxic carriers and 52 patients, which currently integrates the European spinocerebellar ataxia type 3/Machado-Joseph disease Initiative (ESMI), a large European longitudinal MJD cohort. Moreover, we summarize the main studies based on this cohort and highlight the contributions made to advances in MJD research. Knowledge of the profile of the Azorean MJD cohort is not only important in the context of emergent interventional trials but is also pertinent for the implementation of adequate interventional measures, constituting relevant information for Lay Associations and providing data to guide healthcare decision makers.

## 1. Machado-Joseph Disease: Overview

Machado-Joseph disease (MJD)/spinocerebellar ataxia type 3 (SCA3) is a currently untreatable autosomal dominant multisystem neurodegenerative disease of adult onset. Although comprehensive epidemiological data is unavailable worldwide, MJD is thought to be the most common spinocerebellar ataxia [1], reaching a prevalence of 3.1 per 100,000 people in Portugal [2]. In the Portuguese archipelago of the Azores, an epidemiological survey identified an overall prevalence of 39 per 100,000 individuals (1/2544), with values as high as 1 in 158 in Flores Island [3].

Suggestive findings of MJD include the presence of a family history of progressive cerebellar ataxia, which is associated in variable degrees with other neurological alterations, such as pyramidal signs, a dystonic-rigid extrapyramidal syndrome, significant peripheral amyotrophy, and generalized areflexia [4]. Minor, but more specific features, such as external progressive ophthalmoplegia, intention fasciculation-like movements of facial and lingual muscles, as well as bulging eyes, may also be of importance for the differential diagnosis (reviewed in [5]). Neuropathological complexity is characteristic of MJD, and almost invariably the most severely affected are the substantia nigra and the dentate nucleus of the cerebellum [6]. Average age at onset (AO) has been reported as being around 40 years old, its major determinant being the number of CAG motifs at exon 10 of MJD’s causative gene, *ATXN3*. The inverse correlation between the number of CAG repeats and AO is, however, incomplete, with ~30% to ~50% of the variance in onset not being explained by the length of the CAG tract itself. Efforts to identify factors that can explain the amount of variation in AO left unaccounted by the causative mutation have resulted in the proposal of several genetic modifiers (e.g., [7,8,9,10]), whose clinical utility, however, remains to be established.

*ATXN3* is located at 14q32.1 [11] and spans a genomic region of approximately 48 kb [12]. In its wild-type form, *ATXN3* contains from 13 to 44 CAG repeats [13] and encodes the native ataxin-3 protein, a cysteine protease with deubiquitinating activity that is widely expressed in neuronal and non-neuronal tissues (reviewed in [14]). Native ataxin-3 participates in protein quality control pathways, assisting with proteasomal targeting of several substrates [15]. The mutated form of the *ATXN3* gene, usually present in heterozygous MJD patients, exhibits an expanded CAG tract surpassing 60 CAG repeats (reviewed in [5]), which translates into an abnormally elongated polyglutamine protein that gains a neurotoxic function, is prone to aggregation, and interferes with cellular homeostasis, initiating a cascade of pathogenic events, including disturbance of important cellular systems, such as transcription, autophagy, and mitochondrial function [16].

The identification of the *ATXN3* gene allowed the molecular testing of patients with the aim of confirming a clinical diagnosis; moreover, testing of healthy at-risk individuals in the context of the pre-symptomatic test also became possible (reviewed in [17]). Noteworthy, preclinical carriers and MJD patients in the earliest phases of the disease are of great value for better understanding the timeline of disease-related events. Also, upon the identification of disease-modifying agents, intervention should start in the preclinical stage of disease, in which neuropathological changes are starting (reviewed in [18]).

Notwithstanding the pleotropic nature of MJD, which involves several neurological systems, cerebellar alterations are usually considered the most relevant, and gait ataxia constitutes the first symptom in ~85% of the patients [19]. Several rating scales have been developed to capture ataxia severity, namely the Scale for the Assessment and Rating of Ataxia (SARA; [20]). SARA, whose score ranges from 0 to 40 (highest degree of ataxia severity), is an eight-item scale that includes common clinical exam items performed as components of routine assessments of cerebellar impairment [21]: gait, stance, sitting, speech disturbance, finger chase, nose-to-finger test, fast alternating hand movements, and heel-shin slide are included. The SARA score can be further decomposed into several sub-scores, namely axial, appendicular, and upper limb [22]. Because SARA does not address non-ataxic manifestations, additional scales need to be simultaneously used in the evaluation of patients (reviewed in [23]). The Inventory of Non-Ataxia Signs (INAS) is one of such scales; INAS allows one to determine the presence and severity of non-ataxia signs in a standardized way [24], rating several neurological signs including, amongst others, spasticity, tremor, and dystonia. In addition to clinical scales, functional tests, such as the Composite Cerebellar Functional Severity Score (CCFS) [25], are frequently included in studies of the natural history of hereditary ataxias, namely MJD [26]. The CCFS was proposed as a single composite functional score for evaluating cerebellar ataxia in the upper limbs; its score is calculated using the time to perform the nine-hole peg test (9HPT) and the click test [25]. Importantly, the CCFS score, which is adjusted for age, correlates with the impression of patients concerning their own disease severity [27].

Nonmotor symptoms have been widely described in MJD patients and include sleep disturbances involving excessive daytime sleepiness, due in most cases to impaired nocturnal sleeping [28,29]. Moreover, certain sleep-related disorders, such as restless legs syndrome (RLS), a condition clinically characterized by an urge to move the legs, have also been described in MJD (e.g., [30]). The impact of sleep disturbances in ataxia has, in several cases, been studied using the Pittsburgh Sleep Quality Index (PSQI) (e.g., [31]), which is considered a robust and reliable tool for the evaluation of sleep quality and its disturbances, both in clinical practice and in research activities. PSQI [32] is a self-rated questionnaire that evaluates sleep quality over a 1-month time interval; PSQI has already been applied to MJD patients with the aim of increasing knowledge on the impact of sleep disturbances on disease manifestations (e.g., [33]). Depressive symptoms have also been reported in MJD patients; indeed, the presence of moderate-to-severe depressive scores in nearly 34% of MJD patients has been described [34]. Depression scores have been shown to correlate with motor impairment in MJD patients, but whether depression is more reactive and not primarily related to the disease process is a matter of debate (e.g., [35]), reinforcing the need for studies with a larger number of patients. Instruments such as the Patient Health Questionnaire-9 (PHQ-9), a nine-item depression scale that is used as a screening tool for depression, can aid in studying the prevalence and understanding the influence of depressive symptoms on the overall clinical picture of MJD patients [35].

The myriad of motor and non-motor symptoms characteristic of MJD impact the quality of life of patients, limiting mobility, self-care, psychological well-being, and ability to perform day-to-day tasks. disease Therefore, the thorough evaluation of disease requires that effects on quality of life are also determined; this allows for the full capture of the disease spectrum and to establish the relevance for patients of changes in clinical rating scales. To tackle this need, patients’ reported outcomes (PROMs) are being increasingly used in ataxia research. The activities of daily living (ADL) questionnaire, originally part of the Friedreich ataxia rating scale (FARS) [36], is now frequently applied in observational and interventional studies of ataxia (e.g., [37,38]). Additional patient-reported quality of life metrics can be derived from EQ-5D-3L [39]. Although its value in longitudinal studies of ataxias is questionable [40], EQ-5D-3L can be used to provide a generic measure of health-related quality of life, including the EQ-5D descriptive system and the EQ visual analogue scale (EQ VAS). EQ-5D comprises the annotation of problems in the dimensions of mobility, self-care, usual activities, pain/discomfort, and anxiety/depression [39].

Increased understanding of the pathogenic processes that lead to disease upon the presence of the mutated *ATXN3* gene has been providing clues to potential therapeutic targets, and interventional trials built upon such targets have already begun. Detailed knowledge of MJD cohorts should provide crucial information towards the implementation of interventional measures, generate information that can be shared with Lay Associations and provide data to guide healthcare decision makers, both at the global and regional levels. Moreover, clinical, genetic, and biochemical characterization of MJD clusters will favor several aspects of future interventional trials, such as better stratification of patients. As a worldwide rare disorder, MJD trials are unfeasible unless the characterization of cohorts of patients is timely planned. The organization of multicentric international consortia with the aim to prepare trial-ready cohorts of MJD, such as the European SCA3/MJD initiative (ESMI), provides the adequate setup for well-powered collaborative interventional studies.

## 2. The Azorean MJD Cohort: Characterization

MJD was first reported as affecting North American individuals of Azorean ancestry, living in the United States [41,42,43]. The common ancestry of the three families initially described (Machado, Thomas, and Joseph, respectively) has supported the interpretation that this would be an “Azorean disease of the nervous system” [44], originating in the islands of the Azores and disseminated by the Portuguese sea travels of the late 15th and 16th centuries [45]. The hypothesis of an Azorean origin of the MJD mutation was dismissed after the study of extended haplotypes in a large setup of MJD patients, leading to the conclusion that the worldwide-spread mutation probably occurred first in Asia and was later diffused throughout Europe; its high prevalence in Portugal could be explained by a founder effect [46].

The initial studies targeting patients from the Azorean MJD families were undertaken by Portuguese neurologists Paula Coutinho and Corino de Andrade [47]. The study of the Azorean MJD cohort was formally initiated in 1992 by a regional multidisciplinary team coordinated by Jorge Santos (MD), Head of the Department of Neurology of the Hospital of Ponta Delgada, São Miguel, Azores. The study of the MJD families was therefore continued through the funding of several research projects. In 2016, the Azorean team of MJD researchers joined, under the coordination of Manuela Lima (University of the Azores), the European SCA3/MJD initiative—the ESMI project—which was centrally directed by Thomas Klockgether, at DZNE (German Center for Neurodegenerative Diseases, Germany). ESMI, in addition to the University of the Azores/Fundação Gaspar Frutuoso, involved five other European research institutions internationally recognized for their work in the field of ataxias (e.g., [20,48,49,50,51]). From 2017 to 2020, the ESMI consortium developed and implemented a harmonized common protocol for the characterization of carriers of the *ATXN3* mutation, including patients and pre-ataxic (PA) subjects. Since 2020, the ESMI project has continued its research activities under a newly formed consortium, the ESMI Network.

The Azorean cohort described in the present work refers to all Azorean MJD mutation carriers that have participated/are actively participating in ESMI, with the corresponding baseline data compiled by 31 October 2022, and will be referred to in this work as the “Azorean MJD cohort”. Although not all MJD subjects in the Azores have been recruited to ESMI, the number of participants should constitute a representative fraction of the total number of carriers of the MJD mutation originating in and currently living in the Azores.

### 2.1. Demographic Characterization

**Figure 1:**
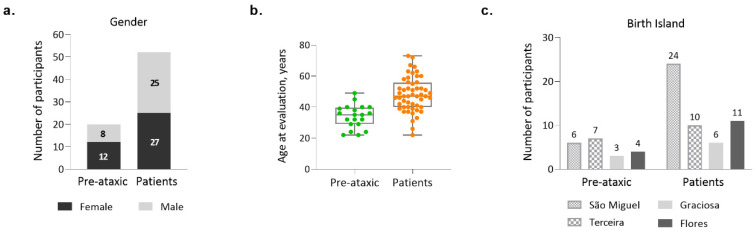
Demographic characterization of the Azorean MJD cohort (n = 72). (**a**) Number of pre-ataxic carriers and patients, by gender; (**b**) Age at evaluation/recruitment for pre-ataxic carriers and patients; (**c**) Number of pre-ataxic carriers and patients, by island of birth.

The Azorean MJD cohort comprises 72 clinically and molecularly confirmed carriers of the *ATXN3* mutation, 35 men and 37 women, living in the Azores archipelago. Noteworthy, 72.2% of the participants are patients, and 27.8% are PA carriers of the MJD mutation (Figure 1a). PA carriers are defined as mutation carriers who present a SARA score lower than 3 (Maas et al., 2015); they were voluntarily enrolled in ESMI after performing a pre-symptomatic test via the regional health system and being aware of their genetic status. The average age at recruitment of the total cohort was 44.2 years (±12 years—standard deviation—SD), ranging from 22 to 73 years of age. The average age at evaluation (recruitment) was distinct between PA carriers and patients (Mann Whitney test, *p* < 0.0001), with patients being older, as expected (Figure 1b).

Subjects from the Azorean cohort originate from the islands of São Miguel, Terceira, Graciosa, and Flores (Figure 1c). Noteworthy, whereas São Miguel, the most populated of the Azorean islands, is the one with the highest number of recruited patients, Terceira Island individually contributes the largest number of PA carriers to the total Azorean cohort.

### 2.2. Genetic Profile of the CAG Repeat at the ATXN3 Gene

**Figure 2:**
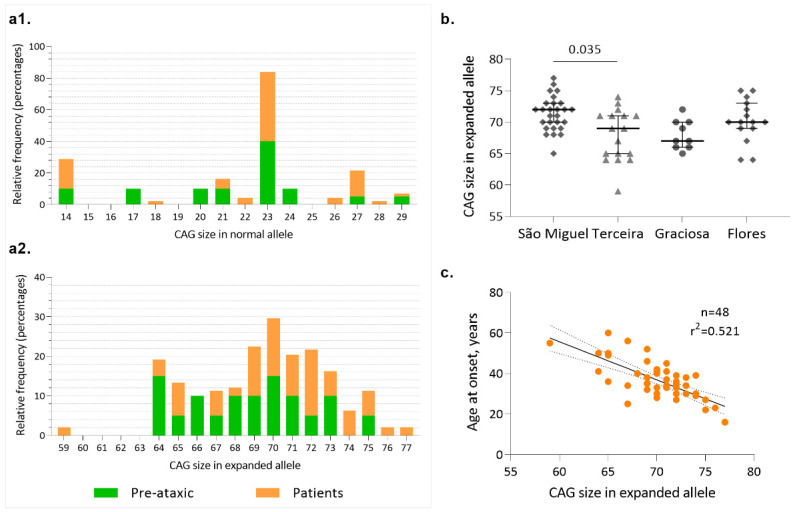
Allelic profile of the CAG repeats at *ATXN3*. (**a1**) Allelic distribution of the normal *ATXN3* alleles (n = 69); (**a2**) Allelic distribution of expanded ATXN3 alleles (n = 69); (**b**) Distribution of the number of CAG repeats at the MJD locus by island of birth of carriers; (**c**) Linear relationship between the CAG number in the expanded allele and age at onset in MJD patients.

**Figure 3:**
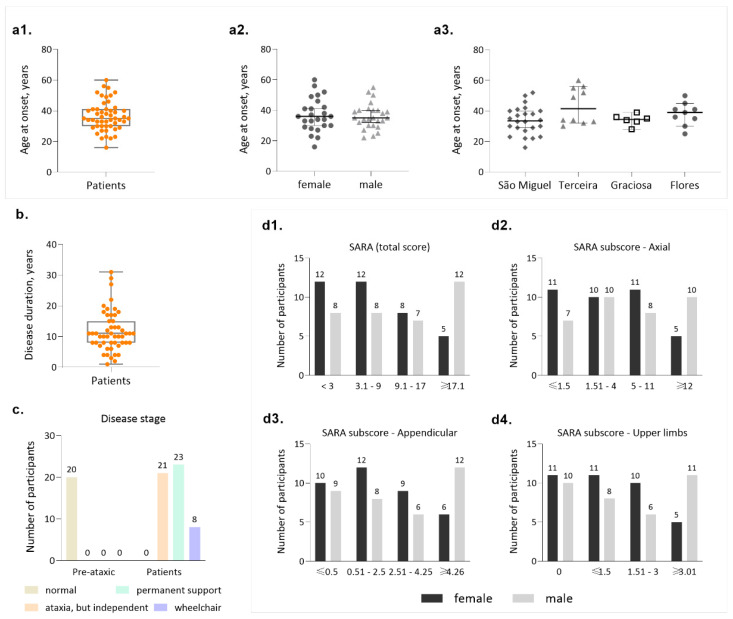
Main clinical features of the MJD Azorean cohort. (**a1**) Distribution of age at onset in MJD patients; (**a2**) Distribution of age at onset by gender; (**a3**) Distribution of age at onset by island of birth of the patients; (**b**) Disease duration in patients; (**c**) Frequencies of disease stages, as proposed by Klockgether and collaborators (1998); (**d1**) Distribution of patients by categories of the total SARA score (maximum score = 40); (**d2**) Distribution of patients by categories of the axial subscore of SARA (maximum subscore = 18); (**d3**) Distribution of patients by categories of the appendicular subscore of SARA (maximum subscore = 12); (**d4**) Distribution of patients by categories of the upper limbs subscore of SARA (maximum subscore = 12).

Allelic distribution of the CAG repeats at wild-type and expanded chromosomes of MJD carriers is shown in Figure 2(a1,a2), respectively. In wild-type chromosomes, the most frequent allele contains 23 repeats, both in PA carriers and patients, in accordance with previous studies that have analyzed ~2000 wild-type chromosomes from the Portuguese population [13]. In the expanded chromosome, alleles with 72 repeats were the most frequent in patients, whereas in PA carriers, a bimodal distribution was observed, with alleles 64 and 70 as the most frequent (Figure 2(a2)). The distribution of the mutated alleles, taking into consideration the island of birth of the MJD subjects (Figure 2b), shows that there are differences between carriers from São Miguel (median of 71 CAGs) and carriers from Terceira (median of 68 CAGs); however, no differences on AO were detected between islands (Figure 3(a3)).

In the Azorean cohort, the number of repeats in the expanded allele explains 52% of the variance of the AO (Figure 2c), a value among the highest reported in European cohorts (e.g., [52,53]). We hypothesize that this “higher” accuracy of the CAG repeats to predict onset in our cohort is related to the continuous follow-up of families, which allows the early detection of carriers [54].

### 2.3. Clinical Features

Onset of symptoms in carriers of the MJD mutation is the most used phenotypic indicator in genotype/phenotype correlation studies. In the Azores Islands, the genealogical structure of the families is well known, and a continuous follow-up has been in place since 1992, potentially implying a high degree of reliability for AO, as mentioned previously. Our cohort presents an average age at onset of 36.5 (±9.55 SD) years, with extremes of 16 and 60 years of age (Figure 3(a1)). Onset is similar for males and female patients (Figure 3(a2)), although both the lowest and highest ages at onset are reported for females. Patients from different islands show similar AO (Figure 3(a3)). Average disease duration is 11.7 years (±6.54 SD), with extremes of 1 and 31 years (Figure 3b). Considering that the survival time for MJD is 21.4 years [55], some of the patients included in this cohort can be considered to have a long disease duration and are predicted to display severe incapacity, although such cases are not frequent in our cohort, as explored further ahead.

MJD carriers in our cohort were classified into the stages of disease defined by Klockgether and collaborators (1998): stage 0 is characterized by the absence of gait difficulties; stage 1 corresponds to disease onset, as defined by the appearance of gait difficulties; stage 2 corresponds to the loss of independent gait; and stage 3 is defined by the confinement to a wheelchair [56]. As expected, all PA carriers can walk independently (stage 0, n = 20, Figure 3c); importantly, a considerable fraction of patients are indeed still able to walk, either independently (stage 1, n =21/52) or with permanent support (stage 2, n = 23/52). Only 8/52 of the patients are permanently dependent on a wheelchair (stage 3, Figure 3c). This distribution of disease stages in our cohort is important in the context of emergent clinical trials, where still being able to walk should constitute an important eligibility criterion [27].

Clinical evaluation of MJD carriers from the Azorean cohort was performed using two rating scales: SARA ([20]) and INAS [24]. The average SARA score was 14.39 (±8.19 SD) ranging from 3 to 29 points (Figure 3(d1)). When considering the categories established based on the total SARA value, most patients (20/52) display a score between 3 and 9 (Figure 3(d1)), further confirming that in this cohort there is a considerable number of patients who are not severely affected. Notably, women make up the majority of patients in the group with the lowest SARA score, whereas men are more represented in the group displaying the highest SARA score (≥17, Figure 3(d1)), although average disease duration in women (9.9 ± 3.8 SD) and men (13.4 ± 8.1 SD) were similar (Mann-Whitney test, *p* = 0.140). When analyzing individually the components of the SARA (Figure 3(d2–d4)), it is possible to observe that all categories are represented in this cohort and that in the category with the highest severity, men are always more represented than women (Figure 3(d2)).

**Figure 4:**
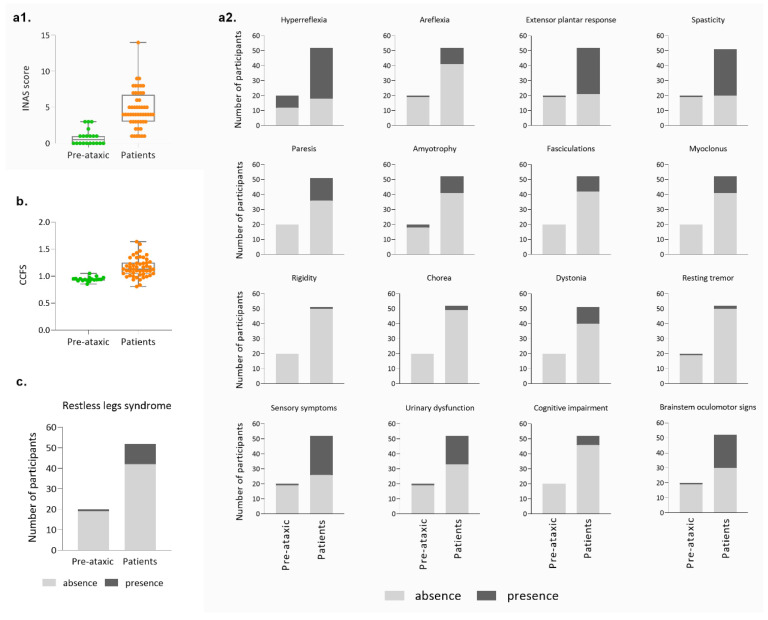
Main clinical features of the Azorean cohort (cont.). (**a1**) Distribution of the total INAS score of pre-ataxic carriers and patients; (**a2**) Presence and absence of the several signs/symptoms evaluated by the individual items of the INAS score; (**b**) CCFS score in pre-ataxic carriers and patients; (**c**) Frequency of restless leg syndrome in MJD mutation carriers.

Non-ataxic signs of MJD patients from the Azorean cohort were evaluated using INAS; the average score in the PA carriers was 0.85 (±1.09 SD), evidencing that non-cerebellar manifestations can be present before ataxia onset, as already described ([19]; Figure 4(a1)). Patients presented an average INAS value of 4.69 (±2.58 SD). Since the INAS maximum score is 16 points, Azorean MJD carriers show a reduced level of affectation on non-cerebellar features. The analysis of individual INAS items shows that pyramidal signs, sensory symptoms, and brainstem oculomotor signs are the most frequent non-cerebellar manifestations. Notably, rigidity and resting tremor are almost completely absent in this cohort (Figure 4(a2)). Although values of the CCFS score calculated separately for PA carriers and patients show some overlap between the two groups (Figure 4b), upper limb coordination was more impaired in patients (1.16 ± 0.18 SD) than in PA carriers (0.94 ± 0.04 SD), as would be expected. Restless legs syndrome was reported in 11 out of the 61 carriers in which this symptom was registered (Figure 4c).

### 2.4. Patients’ Reported Outcome Measures (PROMs)

**Figure 5:**
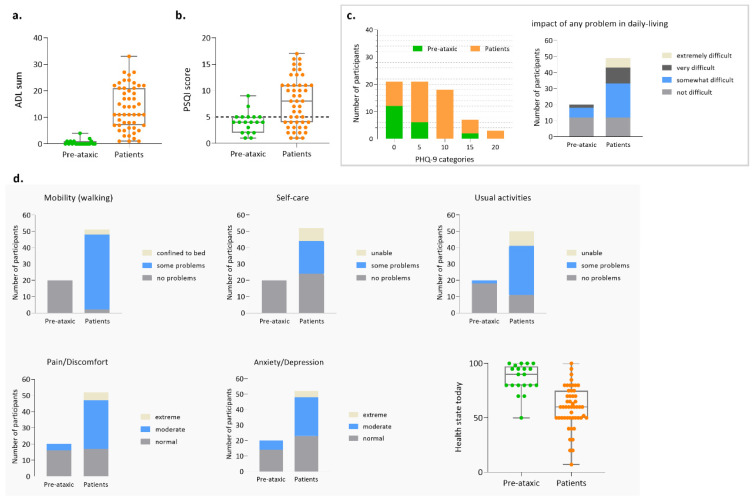
Patient-reported measures for the Azorean MJD cohort. (**a**) Score for the activities of daily living (ADL) questionnaire; (**b**) Score for the Pittsburgh Sleep Quality Index (PSQI); (**c**) Score for the Patient Health Questionnaire (PHQ-9); (**d**) Score for the EQ-5D-3L.

As previously reinforced, patient-based measures of subjective health status are currently considered of great relevance, both at the clinic and for research purposes, namely as endpoints of interventional trials [57]. Activities of daily living (ADL) questionnaire, whose rating produces a score from 0 to 36, indicates, as expected, that patients have functional deterioration (13.5 ± 8.19 SD) of their activities of daily living (e.g., dressing, cutting food, personal hygiene) compared to pre-ataxic carriers (0.5 ± 1 SD) (Figure 5a). Sleep quality was evaluated using the PSQI, which determines sleep quality and perturbances using seven components, producing a total score ranging from 0 to 21; poor sleep quality is indicated whenever the PSQI score is higher than five. In the Azorean MJD cohort, although only two PA carriers have a PSQI score higher than five, most patients (29/48) present poor sleep quality (Figure 5b). Depressive symptoms were evaluated by the 9-item Patient Health Questionnaire (PHQ-9), whose score ranges from 0 to 27 points; scores of 5, 10, 15, and 20 represent cutoffs for mild, moderate, moderately severe, and severe depression, respectively. In PA carriers, the majority of subjects (12/20) presented scores lower than five; noteworthy, 6/20 presented mild depression and 2/20 presented moderately severe depression. In patients, mild depression was present in 15/51 patients; moderate depression was present in 18/51 patients; moderately severe depression was present in 5/51 patients; and 4/51 patients presented with severe depression (Figure 5c). As expected, almost all patients (46/51) reported some problems walking, as indicated by the mobility item evaluation through EQ-5D-3L (Figure 5d). Of note, more than half of the patients reported moderate (30/52) or extreme (5/52) pain/discomfort. In concordance with the results based on the PHQ-9, 6/20 pre-ataxic carriers reported moderate levels of anxiety/depression, as measured by the EQ-5D-3L (Figure 5d).

### 2.5. Relationship between Demographic, Clinical, and Patient-Reported Outcome Measures (PROMs)

**Figure 6:**
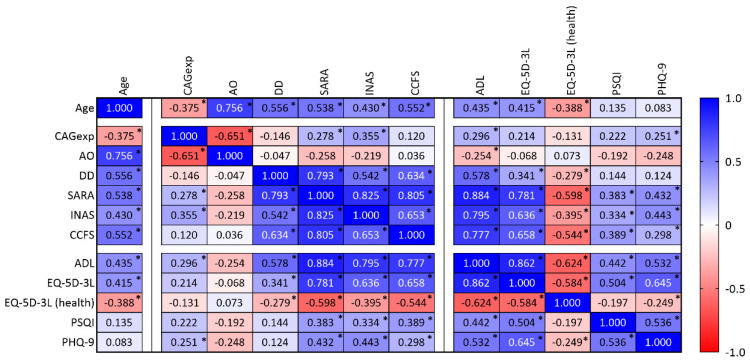
Correlation matrix showing variables analyzed in the present study, namely age, the number of CAG repeats in expanded alleles (CAGexp), age at onset (AO), disease duration (DD), SARA, INAS, CCFS, ASL, EQ-5D-3L (including the health status), PSQI, and PHQ-9. Spearman correlation coefficients (cell values) were shown, and each cell was colored according to its range and direction (positive or negative). * *p*-value lower than 0.05.

Although the primary purpose of this work was to perform a description of the Azorean MJD cohort, some associations between variables could be tested. A relationship between age at evaluation (age) and all clinical variables was found (Figure 6): carriers recruited at an older age show higher disease severity as measured by clinical/functional scales (SARA, INAS, and CCFS). Interestingly, SARA, INAS, and CCFS scores correlate with ADL, EQ-5D-3L, PSQI, and PHQ-9 scores. For example, a strong relationship (rho = 0.884) was found between the ADL score (in which impairment is reported by the carriers) and the SARA score (provided by clinical evaluation), showing that the perception of the individuals concerning their own disease status is coherent with clinical measures (Figure 6).

## 3. The Azorean MJD Cohort: Contribute to Advances in MJD Research

The Azorean cohort has been providing privileged research opportunities in the field of MJD; in fact, this cohort presents several distinctive features: (a) MJD families originate from the Azorean population, which itself has been subjected to a certain degree of isolation and is derived from a limited number of founders (e.g., [58,59]); (b) the genealogical structure of the families is well known, and patients can be traced back into their respective extended families; and (c) the centralized health system has integrated a research program specific to MJD into its health system, thus allowing over the years a detailed follow-up of patients by a reduced number of neurologists and facilitating accurate annotation of disease features and decreasing inter-observational error.

Research on the origins of MJD in the Azores Islands [60,61] provided the foundations for the study of the mechanisms associated with the dispersion of the *ATXN3* gene. In fact, the exceptional prevalence of MJD in the Azorean population was initially postulated to represent the result of a founder effect, as observed for other genetic diseases in similar populations (e.g., [62]). To test this hypothesis, Lima and collaborators [60,61] reconstructed the ascending genealogies of 32 Azorean families, which represented 103 patients, mainly originating from the islands of São Miguel, Terceira, Graciosa, and Flores. Interestingly, no founders common to all genealogies were identified, and their chronological and geographic distribution indicated that more than one MJD mutation had been introduced in the Azores, probably by settlers coming from the Portuguese mainland. This finding was subsequently confirmed by Gaspar and collaborators [45], using molecular data: two distinct disease haplotypes were present in the Azores, one in families originating from São Miguel and the other in families from Flores.

Understanding the factors leading to the high prevalence of an autosomal dominant disease clustering in a population is relevant from several perspectives, including health system planning. Although there are a priori expectations that a gene leading to an incapacitating disease such as MJD could be targeted for negative selection, prevalence values do not seem to support this idea. We have investigated the differential survival, nuptiality, and fertility mechanisms in MJD patients with distinct ages at onset and clinical presentations [63]. The fully documented reproductive histories and known dates of death of a subset of Azorean MJD patients allowed for the conclusion that age at onset and length of survival were associated with marital status, with the married cases having a later mean age at onset and a longer mean survival time [63]. Moreover, clinical presentation was associated with fertility, with significantly fewer children born to the early-onset and more severely affected patients [63]. Segregation distortion is a phenomenon that can also have a potential effect on MJD recurrency in populations, considering that the occurrence of distortion in the transmission, favoring the mutant allele (i.e., the mutant allele is transmitted more than 50% of the time) could justify the high number of carriers. We have studied in the Azorean cohort the transmission behavior of *ATXN3* alleles ([64,65]), concluding that segregation was in accordance with the expected Mendelian proportions, thus ruling out a role for this phenomenon in the prevalence of MJD.

The identification of the *ATXN3* gene led to the availability of a molecular test that could confirm the status of a carrier. In Portugal, a National Program of Predictive Testing and Genetic Counselling (PTGC) for MJD was established in 1996 [66]. In the Azores, prior to the availability of PTCG, we studied the level of knowledge about the disease, the expected level of request for predictive testing, and the intentions of at-risk individuals concerning their reproductive decisions [67]. Upon availability of the test, evaluation of the emotional status of the individuals tested [68] revealed that, although at short term the test result did not cause a decrease in psychological well-being, in the long term [69] moderate to severe stress levels were present in most of the individuals which, 5 years prior, received an unfavorable result in the predictive testing. Therefore, these studies drew attention to the need to monitor the impact of the predictive test and develop strategies to minimize psychological deterioration after taking the genetic test [69].

MJD, similarly to other expansion disorders, displays several “non-mendelian” features, among which is variable expressivity, manifested by the different combinations of affected neurologic systems, by inter-individual differences in rates of disease progression, or by the heterogeneity in age at onset, which remains the best studied readout of variable expressivity. Several mechanisms related to the causative gene itself, *ATXN3*, have been investigated in the Azorean MJD cohort to further understand the variable expressivity of the disease. Since alternative splicing (i.e., the regulatory mechanism that enables one gene to produce multiple mature transcripts with different sequences) is a mechanism that occurs in most human genes (reviewed in [70]), we have analyzed the occurrence of alternative splicing at the *ATXN3* gene [70]. After assessing the impact of the variation of transcripts putatively translated on the integrity/disruption of the ataxin-3 domains, we have been able to classify some transcripts as “protective” (i.e., lacking the CAG tract), whereas others could be more “toxic” displaying a polyalanine instead of a polyglutamine tract [70]. A more thorough study of alternative splicing patterns of the *ATXN3* gene is currently being conducted in our group (Raposo, M., personal communication). Within the *ATXN3* gene, regulatory regions (5 prime and 3 prime untranslated regions) hold the potential to modulate expression. We have therefore assessed the variation in *ATXN3* UTRs using blood samples of molecularly confirmed MJD patients from the Azorean cohort and predicted their functional impact, identifying variants whose presence/absence contributed to modulation of onset ([71,72]). The incompleteness of the explanation of onset provided by the CAG tract also prompted the search for other genes whose variation could modulate onset, in addition to *ATXN3* itself—genetic modifiers. Some of the studies that we have performed analyzed candidates selected based on their previous association with other neurodegenerative diseases (e.g., [7]) or because they corresponded to genes involved in molecular pathways known to be affected in MJD (e.g., [73,74]). The size of repeats at other (CAG)n disease loci (e.g., [52,54]) and allelic variants at the apolipoprotein E (APOE) and interleukin 6 loci (e.g., [7,74]) are among the modifiers that the Azorean MJD cohort helped identify. The success of the quest for MJD genetic modifiers that we and others have performed has been limited, with the identification of single genes showing a modest impact on onset and a large part of it still lacking functional verification. Moreover, replicating in independent populations the effects of many of such modifiers has been challenging; indeed, as stated by Raposo and collaborators [54], the ability to replicate findings referring to MJD genetic modifiers is dependent on how comparable the replication cohorts are relative to the discovery studies, both in the allelic profile of the causal mutation (CAG tract size in polyglutamine SCAs) as well as in the allelic frequencies of the candidate modifier locus/loci. Unbiased genome-wide association studies (GWAS), which are increasingly available, are expected to aid in the identification of MJD modifiers. Indeed, profiting from the extended genealogies available for the Azorean MJD families, we performed whole-exome sequencing in a discovery cohort of age at onset concordant and discordant first-degree relative pairs of Azorean patients. We aimed to identify candidate variants whose genotypes differed for each discordant pair but were shared in each concordant pair [10]. This study allowed the identification of 18 disease-modifying pathways; noteworthy, variants at *PARD3*, *NFKB1*, *CHD5*, *ACTG1*, *CFAP57*, *DLGAP2*, *ITGB1*, *DIDO1,* and *CERS4* were shown to modulate age at onset in MJD, adding between 1.6% and 10% to the explanation of the variation in AO [10].

While MJD remains without treatment, compounds have already begun being tested in the context of clinical trials. Given the global rarity of the disorder on one hand, and its slow progression on the other, demonstrating an effect for any compound will require sensitivity measures of disease progression that are able to detect therapeutic benefits. As clinical scales might not be sensitive enough to be used exclusively, numerous studies have been carried out to identify molecular biomarkers of MJD (reviewed in [18]). Mutated ataxin-3 exerts its effects in several cellular pathways, as previously descrubed [75]; alterations identified in such pathways could constitute potential biomarkers of disease progression (reviewed in [18]). Transcription is one of the mechanisms compromised in MJD patients and seems to be affected via the recruitment of transcription factors into polyQ-rich inclusions and abnormal interactions with transcriptional factors and co-activators (reviewed in [18]). Indeed, using MJD carriers from the Azorean cohort, we have reported several genes that present differential transcription patterns (i.e., abundance or depletion of transcripts) in patients as compared to controls [76], which constitute candidate transcriptional biomarkers of MJD. We have further provided evidence that peripheral blood cells from MJD carriers show changes in the mitochondrial apoptotic pathway [77], indicating a higher susceptibility to the apoptotic stimulus in MJD patients.

In summary, progress in understanding of epidemiological and molecular aspects of MJD derived from studies with Azorean patients demonstrate the importance of this cohort in the global landscape of this disease; it is expected that Azorean patients and families will be also crucial to emerging clinical trials.

## Data Availability

The data presented in this study are available on request from the corresponding author.
